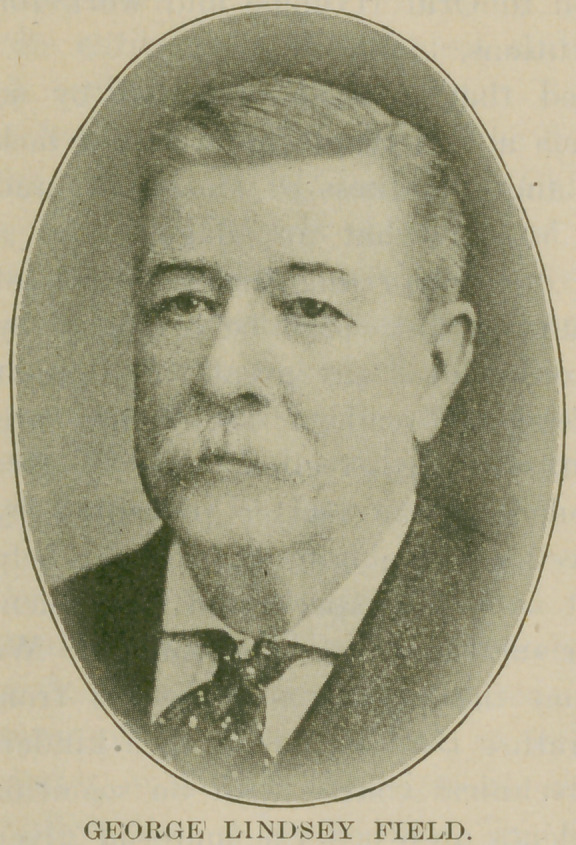# Dr. George Lindsey Field, D.D.S.

**Published:** 1916-11

**Authors:** 


### OBITUARY

GEORGE LINDSEY FIELD.

Dr. George Lindsey Field, D.D.S. Dr. Field was born in England April 19, 1835, and died in Detroit, October 28, 1916. His death was indirectly due to a broken hip from being struck by a street car. He began his dental career as an indentured apprentice in the office of Dr. C. W. Spalding, of St. Louis, Mo., in the fall of 1851, when only sixteen years of age. After three years he associated with Dr. H. J. McKellops, of St. Louis, as a laboratory assistant. He began the practice of dentistry for himself in Huntsville, Mo. but remained in that place for only a few months. He returned to Detroit, the home of his boyhood, in 1856, and started a practice under the handicap of no money or other helpful influence. He had acquired a good degree of technical skill during and after his apprenticeship, and after a tedious struggle to get established, he took the place that was due his genial and attractive personality. He had studied with men of character and high ideals of professional practice. Naturally he took this attitude with his patients, and their confidence in his ethical standards soon brought him a select and appreciative clientele. He acquired a large and lucrative practice for his day and time. He was a genial and courteous gentleman and always had a strong social position in the city of Detroit and he had a host of warm friends everywhere he was known.

The position he took in the social and civic life of Detroit, he also took in his profession. He was an ardent lover of his profession and was always found lending his influence and powers to develop every proposition for the improvement of the profession technically and professionally. He helped to organize the State Dental Society and the Detroit local, as well as many of the district societies. He gave liberally of his time and talents to promote dental society work. He always attended the state and national meetings and did much to make the programmes of these meetings interesting and instructive. He was president of the Michigan State Dental Society for three years, and was president of the Detroit Dental Society for three years. He attended the national and state meetings regularly until his health failed and he became physically unable to attend any meetings He was Professor of Clinical Operative Dentistry in the Dental Department of the Detroit College of Medicine for the entire time of its existence, about fifteen years. He was always interested in the Dental Department of the State University and was largely instrumental in its establishment. He was respected, honored and loved by his professional conferees. When he retired from practice after fifty-six years of active practice in Detroit, the Detroit and Michigan dentists gave him a testimonial banquet and presented him a silver "loving cup." He was an active member of Delta Sigma Delta Fraternity. He is survived by his wife, and a married daughter, Mrs. John R. Campbell, 80 W. Hancock Ave., Detroit.

## Figures and Tables

**Figure f1:**